# Dr. Ware on the Natural History and Treatment of Delirium Tremens

**Published:** 1847-04

**Authors:** John Ware


### IV. THE NATURAL HISTORY AND TREATMENT OF DELIRIUM TREMENS.

BY JOHN WARE, M.D.

[Delirium tremens is one of those acute diseases, the natural progress—in other words, the Natural History of which is generally complicated and obscured, or at least rendered doubtful, by the effects of the treatment most commonly had recourse to in this country, viz. that by opium. It has been, therefore, thought a matter of practical importance to give an account of this very interesting and often dangerous malady, as it shows itself uninfluenced by treatment. The admirable Memoir* from which the following extracts are taken, was published in Boston in the year 1831; and the reader will find the principal data on which its conclusions are founded, in our Number for January, 1839, p. 268. We are by no means prepared to assert that opium is, in no case, beneficial in delirium tremens; or that it will not sometimes procure sleep when this would not ensue spontaneously; but we are certain, from personal experience, that the opiate treatment has often failed, that it has been occasionally very injurious, and that the sleep which ushered in restoration has frequently, at least, been the mere concomitant or sequent of the medicine, and not its effect. At any rate, it is an essential element in the philosophical knowledge of the pathology and treatment of every disease, to be aware of its natural course, progress, and result.]

Although delirium tremens occurs in various states of the constitution, and in various diseases, and is to be looked upon as a possible event in almost all cases of indisposition among drunkards, yet there is a remarkable similarity in the phenomena presented by the affection, and in the course of symptoms through which it passes, whatever may have been the original state of constitution or disease from which it has proceeded. Its approach is often indicated by the existence of certain symptoms from the very commencement of indisposition. It is particularly likely to take place in those who have suffered from irritability of the stomach and frequent vomiting. Indeed, it often makes its appearance after having been preceded by no other symptom of disease, and comes on as soon as the vomiting ceases. There is commonly also in the beginning of those cases in which delirium finally ensues, a tremor of the hands and limbs, and more frequently of the tongue; a tremulousness of voice producing some indistinctness of articulation; a general anxiety; a hurried manner of moving and speaking; imperfect and disturbed sleep; and startings and twitchings of the limbs. These signs are by no means infallible. They are sometimes observed where delirium does not follow. But where they exist from the very first, are not diminished by the treatment adopted, and do not leave the patient with the other symptoms of his complaint, an attack of delirium tremens may be reasonably expected.

But, on the other hand, it frequently happens that the attack is not indicated by any such symptoms in the early history of the case. The patient appears to be getting on perfectly well, and the original disease to be subsiding in a satisfactory manner, when suddenly it becomes manifest that an attack of delirium tremens is threatened. In either case, however, whether there have been any premonitory symptoms or not, the disease follows very much the same course. The patient first complains that he has not slept well, that he has been disturbed all night by unpleasant dreams, that he has been hard at work, but that matters have not gone right, and his concerns have troubled and perplexed him. During the next day, perhaps, he is tolerably comfortable, has some appetite, moves about his house or place of business; yet he is uneasy and restless, and exhibits those appearances which have been already

* Remarks on the History and Treatment of Delirium Tremens. From the Transactions of the Massachusetts Medical Society. 8vo, pp. 61.

described as indicating the approach of the disease. This continues for one or two days; each night being worse than the preceding, whilst in the day there is an increase of the anxiety, restlessness, and trembling of the limbs, tongue, and voice.

The night is then passed with only one or two short naps, from which the patient awakes with some strong impression upon his mind, of the fallacy of which it is difficult or impossible to convince him. His sleep has been filled with dreams of dangers and perplexities and annoyances, innumerable and indescribable. From this state he passes into that of complete watchfulness and delirium. The dreams of his sleeping become the fancies of his waking hours; and in his delirium he conceives himself to be engaged in the same occupations, beset by the same difficulties, and surrounded by the same dangers, that he has described as giving a character to his dreams. In fact it is difficult in many cases to point out the precise time at which the mind passes from the dominion of the conceptions which have been engendered in sleep, to that of those which are the offspring purely of the disease.

At whatever period this state of entire watchfulness and delirium begins, we are to date from it the commencement of what may be denominated *a paroxysm of delirium tremens.* Yet it will sometimes happen, that, on the morning succeeding the night, from the last continued sleep of which we are to date the commencement of the paroxysm, the patient does not exhibit any unequivocal marks of the delirium by which he is affected. The attendants inform us that he has had but little sleep, and has been very *crazy,* but we find him sufficiently rational to give an account of his feelings, and fully aware of whatever is going on about him. Still his aspect and manner are such as to convey to the mind of one accustomed to the disease, the true state of the case, even although there may be no actual exhibition of delirium during the period of the visit.

Most frequently, however, at this time there are occasional wanderings of mind, though not a continued state of delirium. Thus, while sitting by the patient, we perceive his eye become intently fixed upon some remote spot in the room, or outside a window, as if it had been suddenly caught by some remarkable object;—or he will speak in a loud and quick voice, as if making answer to some one who has addressed him from without, or from behind; or he will start up hastily from his seat or from the bed, and run to another part of the room, or to look beneath the bed, as if in pursuit of something. These impressions are, during the early part of the day, evanescent; but in the latter part the delirium returns, and becomes constant. It increases in violence till about the middle of the night, and then diminishes towards the morning.

On the morning of the second day the delirium is still complete, and is not altered in its character; but the patient is milder and more tractable than during the night. He is as fully possessed of the strange imaginations which have entered into his mind; but he is more easily influenced by his friends, and is more amenable to authority. The second night is generally worse than the first, and there is less abatement of the disease in the ensuing or third morning, and in the early part of the day; still there is some alleviation of symptoms, like that of the day before. The third day is passed much in the same way as the second; but if the disease is to have a favorable termination, the delirium of the third night is less violent than that of the preceding, and the paroxysm terminates in sleep, sometimes in the course of the evening or first part of the night, but most commonly not until the latter part of the night or in the morning. When the disease is about to terminate unfavorably, the delirium continues undiminished until the fatal event takes place.

This description has been taken from cases which were left to take their own course, uninfluenced by medicine. In all essential points it will apply to a majority of cases. Still there are many variations in the time of day at which the paroxysm begins and terminates, in its length, and in other particulars, which cannot be included under any general account. Thus its duration is sometimes less and sometimes greater than that assigned to it. Especially it is apt to be prolonged in those who have had repeated attacks, and in one such case I have known it to extend to nearly six entire days.

During the first part of his sleep the patient is generally uneasy and restless, his breathing is irregular, and is sometimes almost like that of a person dying. During the first few hours, he often wakes once or twice, perhaps gets up and renews the exercises of his delirious state, or else takes merely a little drink, but in either case, goes soon to sleep again.

Soon after getting into a sound sleep, the breathing becomes deep, slow, and sonorous; a profuse sweat breaks out, and for a long time the whole body is bathed with it. After six or eight hours the patient awakes tolerably rational, and sensible of what is going on about him, but generally with some impression left on his mind of the imaginary scenes through which he has passed. He continues for the next twenty-four or even forty-eight hours, to sleep during the greater part of the time. At the end of that period, his restoration appears complete, so far as the peculiar symptoms of delirium tremens are concerned; for he may still be the subject of other affections which have preceded the paroxysm, and which remain after it has subsided.

Almost invariably the occurrence of sleep at the close of the paroxysm is indicative of a favorable termination. In some rare cases, however, the patient actually dies after falling asleep, particularly where sleep has been procured by opium; indeed the only cases which I have seen or known, in which the disease has terminated in this way, have been treated by large doses of opium. In such a case no peculiar symptoms indicate a different result from that which we usually promise ourselves when the patient falls asleep, till after sleep has taken place. But then, instead of gradually passing from a disturbed into a more tranquil and natural slumber, he becomes first more unquiet and restless, moans, breathes with difficulty, and falls at length into a state of complete coma, from which he never awakes.

The disease terminates fatally in several other ways. Sometimes the patient is carried off by the sudden accession of convulsions, and this event is particularly to be looked for in those cases which have begun with them. They also occur very unexpectedly in cases which promise favorably, and which have afforded no ground for anticipating them. Sometimes the patient, after continuing the violent exertions of his delirium to the very last moment, without any of the peculiar signs of approaching dissolution, falls back and expires immediately. Sometimes, during the continuance of the delirium, death comes on from the effects of some disease with which it happens to be complicated, and dissolution occurs in the same way that it would from that disease alone.

. . . . . .

There has been much uniformity of opinion among physicians concerning the object to the accomplishment of which the treatment is to be directed during the paroxysm. This object is the procuring of sleep. The absence of sleep is one of the most remarkable symptoms of the disease. When it terminates favorably, it terminates in sleep. It is not without foundation, therefore, that the treatment has had for its primary indication to bring about this termination. The patient, it has been emphatically said, "*must sleep or die.*" There is no doubt that this is true. But may it not have been too hastily concluded from this undeniable position, that sleep must be procured by the assistance of art, or the patient will die? It is possible that the common impression which has been produced on our minds concerning this is erroneous in two points of view: 1, We have concluded that sleep is the cause of the salutary change which takes place in the disease; and 2, that sleep, in whatever way induced, will have the same effect, and that it is therefore to be induced by artificial means.

In order to determine, concerning any disease, what influence our remedies actually exert upon it, we must first ascertain what will be its course and termination if suffered to go through its usual series of changes without the interference of art. This is a point in the history of diseases to which reference should always be had in deciding upon the principles, or calculating the efficacy of the treatment to be employed. This is particularly desirable in those diseases which, like that now under consideration, have but recently become the subjects of medical observation and inquiry.

I have witnessed a considerable number of cases of delirium tremens, in which the patient, after the establishment of the paroxysm, has been left to contend with it, without the administration of any remedy whose tendency was to cut it short, or in any decided way to modify its symptoms. The active treatment has been confined to the period of indisposition preceding the paroxysm ; and after its accession articles of a negative character alone were administered, with the exception sometimes of purgatives. The result has uniformly been, that the disease has gone through that regular course, which has been already described in the former part of this paper, and has terminated in the manner there described, at a period seldom less than sixty or more than seventy-two hours from the commencement of the paroxysm.

The termination in these cases has also been almost uniformly favorable, except where there has been a combination of the delirium with some acute disease in itself dangerous, or where it has appeared in connexion with some fatal chronic malady. This course has been pursued, I do not mean to say without any deviation, but without any deviation which I believe to have essentially affected the result, in about fifty cases of the several classes which have been described; and although several deaths have taken place among them, none are recorded, except among cases, which I have arranged, whether justly or not, in the third and fourth classes.*

It may be stated, in confirmation of the opinion now expressed concerning the natural tendency of the paroxysm to terminate in a spontaneous and salutary sleep at the end of a certain period, that, even in the reports of cases which have been submitted to the public as evidences of the efficacy of various modes of practice, sleep has not actually taken place sooner than it would have done in the natural course of the disease, if the history which has now been given of it be founded on correct observation. In the cases which I have formerly treated with opium, and which have at last terminated well, a salutary sleep has not actually taken place till toward the close of the third day, let the quantity of opium be what it would. I have indeed seen sleep induced by opium at an earlier period, but it was premature ; it passed into a state of coma, and the patient died.

I am satisfied, therefore, that in cases of delirium tremens, the patient, so far as the paroxysm alone is concerned, should be left to the resources of his own system, particularly that no attempt should be made to force sleep by any of the remedies which are usually supposed to have that tendency ; more particularly that this should not be attempted by the use of opium. I do not undertake to say that it can be never right to administer opium for the removal of the paroxysm itself, but I believe it can be rarely necessary, and I have not yet seen a case in which I think that it was.

* Dr. Ware classes his cases as follows:

1. As the disease occurs as an immediate consequence of a particular excess or excesses in persons not otherwise disposed to disease.

2. As the result of habitual intemperance, without any particular or extraordinary excess.

3. As the disease occurs in connexion with other regularly-formed diseases or as the consequence of injuries, and still having the natural tendency to end in sleep.

4. An irregular form, of a recurrent or less permanent type, not ending in sleep, coming on in the course of other diseases.—ED.

